# Attenuation of influenza virus infectivity with herbal-marine compound (HESA-A): an in vitro study in MDCK cells

**DOI:** 10.1186/1743-422X-9-44

**Published:** 2012-02-16

**Authors:** Parvaneh Mehrbod, Aini Ideris, Abdul Rahman Omar, Mohd Hair-Bejo, Sheau Wei Tan, Masoumeh Tavassoti Kheiri, Mansoureh Tabatabaian

**Affiliations:** 1 Institute of Bioscience, Universiti Putra Malaysia, 43400 Serdang, Selangor, Malaysia; 2 Faculty of Veterinary Medicine, Universiti Putra Malaysia, 43400 Serdang, Selangor, Malaysia; 3 Influenza Unit, Pasteur Institute of Iran, Tehran, Iran

**Keywords:** HESA-A, H1N1, Influenza virus, Cytokine, TNF-α, IL-6

## Abstract

**Background:**

The influenza virus is still one of the most important respiratory risks affecting humans which require effective treatments. In this case, traditional medications are of interest. HESA-A is an active natural biological compound from herbal-marine origin. Previous studies have reported that the therapeutic properties of HESA-A are able to treat psoriasis vulgaris and cancers. However, no antiviral properties have been reported.

**Methods:**

This study was designed to investigate the potential antiviral properties of HESA-A and its effects in modulating TNF-α and IL-6 cytokine levels. HESA-A was prepared in normal saline as a stock solution (0.8 mg/ml, pH = 7.4). Percentages of cell survival when exposed to different concentrations of HESA-A at different time intervals was determined by MTT assay. To study the potential antiviral activity of HESA-A, Madin-Darby Canine Kidney (MDCK) cells were treated with the effective concentration (EC_50_) of HESA-A (0.025 mg/ml) and 100 TCID_50_/0.1 ml of virus sample under different types of exposure.

**Results:**

Based on the MTT method and hemagglutination assay (HA), HESA-A is capable of improving cell viability to 31% and decreasing HA titre to almost 99% in co-penetration exposures. In addition, based on quantitative real-time PCR (qRT-PCR) and enzyme-linked immunosorbent assay (ELISA), it was found that HESA-A causes decrements in TNF-α and IL-6 cytokine expressions, which was significant for TNF-α (*p *≤ 0.05) but not for IL-6.

**Conclusion:**

In conclusion, HESA-A was effective against influenza infection through suppressing cytokine expression.

## Background

Influenza virus A, a member of the Orthomyxoviridae family, is one of the most important causes of acute contagious respiratory diseases worldwide. Its infectivity is increasing due to various drifts and shifts of genetic mutations that cause constant alterations of the antibody-targeted surface glycoproteins. This property makes it extremely difficult to develop effective vaccines and specific drugs [1]. Even conventional drugs such as Amantadine and Oseltamivir, that are able to control the entrance and release of the virus from the host cell based on the viral protein structures, are not effective enough and have shown many cases of side effects and drug resistances [2]. So, there have been suggestions in switching to traditional medication for influenza disease treatment. HESA-A is an active natural biological compound from herbal-marine origin, with a general composition of inorganic, organic and aqueous fractions [3]. Previous studies have reported the therapeutic properties of HESA-A against psoriasis vulgaris, breast cancer and choroidal metastasis [4,5]. However, there is no published evidence on its antiviral activity against influenza virus infectivity.

One of the most important factors which contributed to the pathogenesis of influenza infection has been shown to be cytokine dysregulation. Influenza viruses, especially H1N1 and H5N1, cause downstream induction of pro-inflammatory cytokines such as TNF-α and IL-6, that in turn, cause immune system uncontrolled responses that lead to inflammation [6,7]. Therefore, there have been suggestions that anti-inflammatory and immunomodulatory agents could be effective alternatives to vaccines and antiviral agents against influenza. In this study, the antiviral effect of HESA-A against influenza virus infection was evaluated in vitro.

## Results

### Cell viability

MDCK cells viability was determined after different times of exposure to different concentrations of HESA-A through MTT at an optical density of 540 nm. The results showed that HESA-A had no cytotoxic effect on the cells for concentration of up to 0.05 mg/ml. The EC_50 _of this compound was calculated from the MTT results by two-way ANOVA analysis at 0.025 mg/ml, with no significant toxicity on cell viability (Table 1).

**Table 1:** Mean MTT results of treatments with different concentrations of HESA-A

Sample (mg/ml)	Mean ± SD
0.8	0.21 ± 0.21٭
0.4	0.26 ± 0.24٭
0.2	0.33 ± 0.24٭
0.1	0.43 ± 0.25٭
0.05	0.51 ± 0.22٭
0.025	0.69 ± 0.20
0.013	0.73 ± 0.18
0.0	1.00 ± 0.00

Values (averages of 4 independent experiments) showed cytotoxicity of different concentrations of HESA-A at different exposure time (24, 48 &72 hr) on MDCK cells (mean ± SD). CC_50 _and EC_50 _are determined as 0.05 and 0.025 mg/ml, respectively.: ٭Significantly different from values obtained for HESA-A-treated samples compared to untreated sample (*p *< 0.05)

### HESA-A inhibitory effect on influenza virus

**Figure 1:**
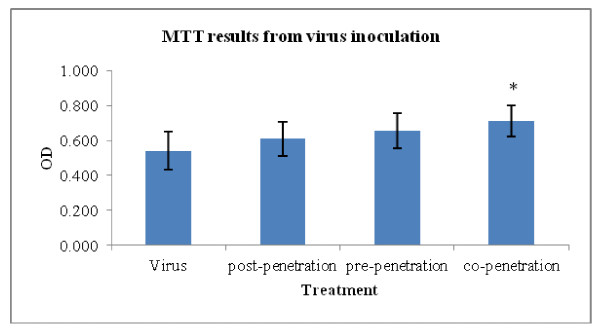
**MTT optical densities for cell viability in response to different types of exposure of HESA-A and virus (averages of 4 independent tests) (mean ± SD)**. *: Significantly different from values obtained for the co-penetration treatment compared to the virus untreated sample (*p *< 0.05).

**Figure 2:**
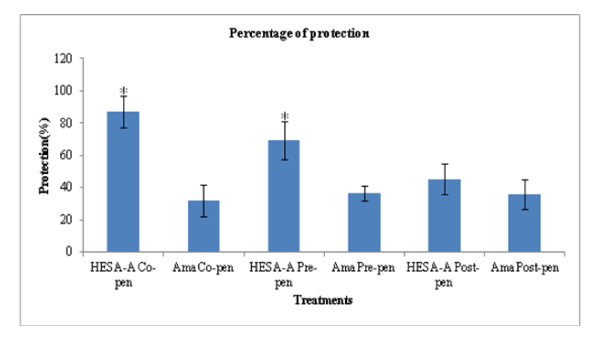
**Comparison of the percent of protection of HESA-A and Amantadine on cell viability**. Data analyzed by SPSS, one way ANOVA are averages of 4 independent experiments (mean ± SD). HESA-A showed a higher degree of protection towards cell viability in comparison with Amantadine treatments. It was significantly higher in co- and pre-penetration treatments. *: Significantly different from values obtained for co- & pre-penetration treatments compared to Amantadine treatments (*p *< 0.05).

In this experiment, there were increments in the optical densities (ODs) measured after running the MTT assay for different exposures, compared with the virus-treated sample without HESA-A. The results for the virus treatment, as well as post-, pre- and co-penetration treatments (mean ± SD) are 0.54 ± 0.11, 0.61 ± 0.10, 0.65 ± 0.10 and 0.71 ± 0.09, respectively. However, as shown in Figure 1, the significant increment in OD (*p *≤ 0.05) was related to the co-penetration exposure. The ODs were also analyzed to examine the percentage of HESA-A protection in combination treatments compared to Amantadine treatments. As shown in Figure 2, the ranges of ODs were significantly higher in all types of treatments, especially the co- & pre- treatments, than the Amantadine-treated samples. In addition, the HESA-A co-penetration treatment showed the highest OD, nearly three times more than the Amantadine co-treatment.

### Hemagglutination assay results

Based on hemagglutination titration, the inhibitory effect of HESA-A on viral activity was shown through a significant reduction in HA titre in all combination treatments. Notably, in the co-penetration treatment, the HA titre decreased by 98.75% compared to the untreated sample (Table 2).

**Table 2:** Hemagglutination assay results evaluating the antiviral activity of HESA-A against influenza virus A

Treatment	Mean ± SD
Virus sample	45.71 ± 15.83
Post-penetration	6.86 ± 11.65*
Pre-penetration	3.43 ± 5.83*
Co-penetration	0.57 ± 1.40*

Values are averages of 4 independent HA assays

*: Significantly different from values obtained for HESA-A-treated samples compared to untreated sample (*p *≤ 0.05)

### Absolute quantification

The HESA-A effects on viral genome load and inflammatory cytokine levels were shown by log_10 _copy number decrements in treatments which were calculated through absolute quantification. Quantitative analysis on the M2 gene of influenza virus A PCR products exposed to HESA for one hour at different types of exposure, showed statistically significant decrements in viral load compared to the virus sample (Table 3).

**Table 3:** M2 Log_10 _copy numbers at different treatments

Sample	Ct (Mean ± SD)	Log_10 _Copy Numbers (Mean ± SD)
Control	0 ± 0	0 ± 0
Virus	10.900 ± 0.026	12.004 ± 0.003
Co-penetration	11.760 ± 0.044	11.916 ± 0.004*
Pre-penetration	11.737 ± 0.055	11.919 ± 0.006*
Post-penetration	11.593 ± 0.445	11.933 ± 0.045*

Log_10 _copy numbers relating to Ct values during amplification of the influenza A H1N1 M2 gene were averages of 2 independent repeats in duplicate and showed significant decrease (*p *< 0.05) in all combination treatments in comparison with the virus untreated sample

For absolute quantification of the cytokine genes, TNF-α and IL-6 PCR product analyses showed considerable decrements in log_10 _copy numbers in all types of exposures. The decrement in TNF-α gene load was significant only in direct exposure of HESA-A to the virus (*p *< 0.05), but not in the pre- & post-penetration exposures, while IL-6 decrements were not significant (Table 4).

**Table 4:** TNF-α and IL-6 log_10 _copy numbers in different treatments

	TNF-α	IL-6
Sample	Log_10 _Copy Numbers (Mean ± SD)	Log_10 _Copy Numbers (Mean ± SD)
Control	10.854 ± 0.051*	11.096 ± 0.137
HESA-A	10.806 ± 0.086*	11.092 ± 0.015
Virus	11.436 ± 0.051	11.464 ± 0.031
Co-penetration	11.244 ± 0.005*	11.104 ± 0.025
Pre-penetration	11.294 ± 0.007	11.258 ± 0.045
Post-penetration	11.447 ± 0.001	11.397 ± 0.397

Log_10 _copy numbers were obtained from Ct values during the amplification of the TNF-α and IL-6 cytokine genes. Data are averages of 2 independent repeats in duplicate

*: significantly different compared with the virus untreated sample (*p *< 0.05)

### Cytokine detection using ELISA assay

TNF-α and IL-6 protein levels in the supernatants of MDCK cell cultures at 24 and 48 hour after exposure were not detected (data not shown), but after 72 hour of treatments, virus-treated samples show significantly higher expression than HESA-treated supernatants. The data was analyzed using One-way ANOVA followed by Tukey post-hoc test (Table 5).

**Table 5:** TNF-α and IL-6 protein concentrations in MDCK culture supernatants (pg/ml) after 72 hr exposure

	TNF-α	IL-6
Treatment	Concentration (Mean ± SD)	Concentration (Mean ± SD)
Negative control	3.94 ± 0.01	2.33 ± 0.00
HESA-A	2.58 ± 0.00	1.33 ± 0.00
Virus	12.88 ± 0.00*	21.67 ± 0.01*
Co-penetration	0.45 ± 0.00	2.00 ± 0.00
Pre-penetration	3.03 ± 0.01	4.00 ± 0.00
Post-penetration	3.64 ± 0.00	5.00 ± 0.00

Concentrations of TNF-α and IL-6 proteins, as determined by ELISA, are expressed as pg/ml (N = 6-8) after a 72 hr incubation time,

*: significantly different from mock-inoculated and treated cells in different experiments (*P *< 0.05)

## Discussion and conclusion

The influenza virus causes severe respiratory diseases that remains as a leading source of annual morbidity [8]. The discovery and development of novel anti-influenza compounds, preferably of natural origin, are required to prevent and treat potential influenza pandemics. Existing therapeutic antiviral agents have limited clinical efficiency with many toxic side effects, but antiviral compounds of natural origin are more easily available and are mostly nontoxic [9,10].

It has been shown that an influenza infection triggers the induction of pro-inflammatory cytokines which activate GTPase proteins by isoprenylation and begins the process of recognizing ssRNA [11] to express the target genes for cytokines, such as IL-1, IL-6, TNF-α and IFN-γ. However, the increased level of pro-inflammatory cytokines after an influenza virus infection, which is caused by an over-responsive immune system, sometimes causes hypercytokinemia. Hypercytokinemia is the systemic expression of a strong immune system and is a potentially lethal reaction which consists of a positive feedback between cytokines and immune cells that, in turn, causes lung inflammation [7,8,12,13]. The most important pro-inflammatory cytokines that are responsible for these reactions are IL-6 and TNF-α. These cytokines are pleiotropic inflammatory cytokines that play important roles in metabolism, apoptosis, massive systemic effects and inflammation, which are hallmarks of influenza [14-16].

HESA-A is a natural compound of herbal-marine origin which contains inorganic, organic and aqueous fractions with a wide range of therapeutic applications [4,17,18]. In this study, the interaction between HESA-A and influenza virus A/H1N1 was evaluated. HESA-A was not toxic on MDCK cells at up to 0.05 mg/ml. As calculated from the MTT results by two-way ANOVA test, the EC_50 _of this compound was obtained at 0.025 mg/ml, with no cytotoxic effect on the cells. The optical densities obtained from the MTT assay showed that co-penetration and pre-penetration treatments had 86.92% ± 9.79 and 69.17% ± 11.8 of protection, respectively. These treatments were more protective (*p *≤ 0.05) against viral cytopathic effects in comparison with the other exposures, even with different Amantadine treatments. Meanwhile, HA results showed a significant decrease in HA titre in all combination treatments compared to the positive control of a virus-treated sample. From these results, it is postulated that HESA-A may interfere with viral membrane fusion by inhibiting penetration or adsorption through HA glycoprotein interference.

The antiviral effects of HESA-A on influenza viral load and cytokine levels in MDCK cell cultures were analyzed using quantitative real-time PCR assay. This technique, which provides absolute copy numbers of the template, is a reliable and more accurate tool compared to relative quantification [19,20]. In this assay, the quantity of targeted genes can be evaluated with reasonable accuracy by using a reliable standard [21]. A standard curve, constructed from standard concentrations (data not shown), was used to determine the copy numbers of target genes related to the Ct value. Significant increments in the cycle thresholds (Cts) of M2 PCR products were observed once HESA-A was applied in all types of combination treatments. The log_10 _copy numbers, which were calculated from the concentrations against mean Ct values, confirmed these significant differences, especially when HESA-A was applied in the co-penetration treatment. In HESA-A and virus combination treatments, TNF-α and IL-6 copy numbers, which were calculated from the standard curves, showed some decrements. The standard curves were obtained by plotting concentrations against mean Ct values. Significantly low expression in TNF-α, but not IL-6, was related to the co-penetration treatment.

The quantification of cytokines by qRT-PCR was also analyzed and confirmed using ELISA. It was found that infection by the virus causes high expression levels of these pro-inflammatory cytokines, while in all combination treatments, the expression of these proteins significantly decreased, especially in co-penetration treatments, which showed 96.51% and 90.77% decrements in TNF-α and IL-6 protein expression, respectively.

In conclusion, it is possible to limit immune system over-expression and turn off lung inflammation caused by influenza infection if proper treatment with HESA-A is applied. Clinical control can prevent this intense cytokine response through early diagnosis and efficient treatment. Further research on the interaction of HESA-A active compounds with different parts of cellular and viral genes are currently underway.

## Materials and methods

### Cell culture and influenza virus propagation

MDCK cells were grown in Dulbecco's Modified Eagle's Medium (DMEM) (Mediatech Cellgro, USA), supplemented with 10% Fetal Bovine Serum (FBS) (PAA, Austria) and 1% Pen/Strep (Mediatech Cellgro, USA) at 37°C in a humidified incubator. The media was changed two to three times per week. The influenza vaccine strain, A/New Jersey/8/76 (H1N1), was purchased from American Type Culture Collection (ATCC) with the Reference Number VR-897™. It was propagated in MDCK cells in the presence of 1 μg/ml of Trypsin_TPCK (Tosylamide Phenylethyl Chloromethyl Keton-treated Trypsin) (Sigma, USA) to create the working stock. During antiviral evaluations, media supplemented with FBS was sucked out and the cell was washed with PBS and then it was treated as needed. Then media supplemented with Trypsin_TPCK was added.

### HESA-A preparation

HESA-A was kindly provided by Dr. Amrollah Ahmadi, Tehran University of Medical Sciences, Tehran, IRAN. In brief, it was dissolved in normal saline, shaken for 30 minutes and filtered to become homogenate. Prior to its use, the stock solution (0.8 mg/ml, pH 7.4) was sterilized through 0.22 μm nylon filter membrane and diluted with DMEM [22].

### Cytotoxicity assay

MDCK cells were incubated in 96-well micro-plate (Nunc, Denmark) for 24 hours at 37°C. Two-fold serial dilutions of HESA-A were added to the semi-confluent cells in triplicates and incubated at different time intervals. Colorimetric MTT assay was performed according to Mehrbod et al. (2009) [23]. The absorbance of color in the solution was analyzed at 540 nm with micro-plate reader machine (BioTek EL 800, US) to calculate the 50% cytotoxic concentration (CC_50_), effective concentration (EC_50_) and viability of the cells by two way ANOVA, SPSS.

### Percentage of protection

After one hour of incubation with EC_50 _of HESA-A in different types of exposure (co-, pre- and post-penetration) with 100 TCID_50 _of the virus, the cells were washed with 1X PBS and, then medium supplemented with Trypsin_TPCK was added (100 μl/well). After 48 hours of incubation at 37°C, viabilities of the cells were evaluated by MTT. Meanwhile, the virus titre was determined by HA assay [23].

The percentage of protection of HESA-A was calculated, in Microsoft Office Excel 2010 and SPSS, from the MTT results of mock-infected and infected cells after 48 hours of exposure, using the following formula:

$$\text{Percentage}\;\text{of}\;\text{protection}=\left[\left(\text{ODT}\right)\text{V}-\left(\text{ODC}\right)\text{V}\right]/\left[\left(\text{ODC}\right)\text{M}-\left(\text{ODC}\right)\text{V}\right]\times 100$$

where (ODT)V, (ODC)V and (ODC)M imply the absorbance of the treated sample, the virus-infected control (no compound) and the negative control (no virus and no compound), respectively [24].

### Hemagglutination assay

To evaluate the presence of the virus in cell culture, in either treated or non-treated samples, serial dilutions of the culture media were added to 96-well U-shape micro-plates. Chicken red blood cells (cRBCs) (0.5%) were added to each well. The assay was carried out as described previously by Mehrbod et al. (2009) [23].

### RNA extraction and generation of cDNA

For each sample, viral RNA was extracted from 200 μl of cell culture fluid using Viral Nucleic Acid Extraction kit II, according to the manufacturer's instructions (Geneaid, Taiwan). The extracted RNA was isolated and re-suspended in 50 μl of sterile RNase-free water.

For cellular RNA extraction, cells were trypsinized and suspended in 1X PBS. After centrifugation, total RNA was purified from the cell pellet using GeneJET™ RNA Purification Kit, according to the procedures (Fermentas, Canada). Extracted total RNA was diluted in 100 μl RNase-free water.

Extracted RNAs, in a volume of 10 μl, were added to each reaction mix of the RevertAid H Minus First Strand cDNA kit (Fermentas, Canada), including random hexamer primers, 5X Reaction buffer, RNase inhibitor and dNTP mix to a final volume of 20 μl. The mix was incubated at 25°C for 5 min, followed by 42°C for 60 min, and terminated at 70°C for 5 min. The concentration of the cDNA templates was measured using the Nanodrop system (Implen NanoPhotometer™ Germany). Virus-inoculated and mock-infected samples were considered as positive and negative controls respectively.

### Primer design

Primers for quantitative real-time PCR (qRT-PCR) were designed and synthesized by First Base Co. Malaysia. The descriptions of the primers used for the amplification of the influenza M2 gene and canine TNF-α and IL-6 genes are as shown in Table 6.

**Table 6:** Primers designed to amplify the M2, TNF-α and IL-6 genes

Name	Primer	accession number	Position	Size
M2-A-For	GGC AAA TGG TAC AGG CAA TG	CY039992	636-655	
M2-A-Rev	AGC AAC GAG AGG ATC ACT TG		760-779	143
TNF-α-For	TGT CAG CTC CAC GCC GTT GG	NM_001003244.4	496-515	
TNF-α-Rev	AGG GAA GAG CTC CCA AAT GGC C		657-678	182
IL-6-For	CTG GGT TCA ATC AGG AGA CCT GCT	NM_001003301.1	353-376	
IL-6-Rev	CGC ACT CAT CCT GCG ACT GCA		578-598	245

### Quantitative real-time PCR

Real-time PCR reactions were performed in total volume of 25 μl, using the CFX 96 Real-Time PCR Detection System (Bio-Rad, USA). Maxima SYBR Green/Fluorescein master mix (2×) (Fermentas, Canada) was used for the amplification. The reaction mixture, which consisted of a final concentration of 1× reaction buffer [KCL and (NH_4_)_2_SO_4_, dNTP, 2.5 mM MgCl_2_, Taq DNA polymerase, SYBR Green dye I and fluorescein passive reference dye], 0.3 μM of each primer and 1 μg/μl of cDNA, was prepared in low-profile 0.2 ml tube strips (Bio-Rad, Hercules, CA, USA). The thermal cycling program for the M2 and TNF-α genes consisted of an initial incubation at 95°C for 3 min, followed by 40 cycles of denaturation at 95°C for 15 s, annealing at 60°C for 30 s and extension at 72°C for 20 s. The amplification conditions for the IL-6 gene was one cycle of initial denaturation at 95°C, followed by 45 cycles of denaturation at 95°C for 30 s, annealing at 58°C for 30 s and extension at 72°C for 20 s. Upon amplification completion, the specificity of the amplified products was confirmed using melting curve analysis, whereby the PCR products were incubated by raising the incubation temperature from 70°C to 90°C with 0.5°C increment per second. All the reactions were performed in duplicates and a mixture containing no cDNA template was used as the negative control. Data acquisition and analysis were performed using the CFX Manager Version 2.0.

### Construction of standards for quantification

For each real-time PCR assay, the cDNA template from the respective positive control sample was diluted 10-fold (10^2 ^copies/μl to 10^7 ^copies/μl) to construct the standard curves. The Maxima™ SYBR Green/Fluorescein qPCR Master Mix Kit (Fermentas, Canada) was used for the amplification assay. Each standard curve was generated by plotting the cycle threshold values (Ct) against the input cDNA copy number (copies/μl) alongside a non-template control (NTC). The concentration of cDNA templates was quantified by the Nanodrop system (Implen NanoPhotometer™ Germany) and the copy numbers for the standards were calculated by the following formula [25]:

$$\text{Number}\;\text{of}\;\text{copies}/\mu \text{l}=\left[6.02\times 10^{23}\left(\text{molecules}/\text{mole}\right)\times \text{DNA}\;\text{concentrations}\;\left(\text{g}/\mu \text{l}\right)]/[\text{Number}\;\text{of}\;\text{bases}\;\text{pairs}\times 660\;\text{daltons}\right]$$

Where 6.02 × 10^23 ^(molecules/mole) is Avogadro's number and 660 daltons is the average weight of a single base pair.

### IL-6 and TNF-α cytokine assays

An enzyme-linked immunosorbent assay (ELISA) was used to examine the effects of viral infection and HESA-A treatments on TNF-α and IL-6 cytokine protein production. Quantitative sandwich ELISA was performed using the Quantikine ELISA kits (R&D Systems, Minneapolis, MN) in micro-plates pre-coated with polyclonal antibodies specific for the IL-6 and TNF-α cytokines. Cell-free supernatants, incubated for 24, 48-72 hours in different treatments, were collected for cytokine analyses and stored at -80°C until further processing. The cultures were repeated on at least four separate occasions, in duplicates. After being washed, the enzyme reaction yielded a blue color that turned yellow when the stop solution was added. The strength of the color relates to the quantity of the cytokines bound in the first step. Finally, the sample values were read off the standard curve.

### Statistical analysis

The data, expressed as mean ± SD, was gathered and analyzed using Microsoft Office Excel 2007 and analysis of variance (ANOVA) (SPSS 18.0). Sample values between different groups and treatments with *p *≤ 0.05 were considered statistically significant.

## Abbreviations

ANOVA: Analysis of variance; ATCC: American Type Culture Collection; CC_50_: Cytotoxic Concentration; DMEM: Dulbecco's Modified Eagle's Medium; EC_50_: Effective Concentration; ELISA: Enzyme-linked immunosorbent assay; FBS: Fetal Bovine Serum; HA: Hemagglutination Assay; MDCK: Madin-Darby Canine Kidney; qRT-PCR: Quantitative real-time PCR; TPCK: Tosylamide Phenylethyl Chloromethyl Keton-treated Trypsin.

## Competing interests

The authors declare that they have no competing interests.

## Authors' contributions

MP, AI, TKM co-defined the research theme. MP designed the methods and experiments. MP carried out the laboratory experiments, analyzed the data and drafted the manuscript. MP, TSW co-worked on the associated data collection and their interpretation. ARO, MP, TSW, TKM, TM and HBM revised the paper critically for important intellectual content. All authors have seen and approved the manuscript.
